# A Previously Healthy Infant with Lemierre Syndrome in the Emergency Department: Case Report

**DOI:** 10.5811/cpcem.1580

**Published:** 2023-08-01

**Authors:** Adeola Adekunbi Kosoko, Omoyeni O. Clement

**Affiliations:** University of Texas Health Sciences Center at Houston, Department of Emergency Medicine, Houston, Texas

**Keywords:** infant, pediatric, Lemierre, thrombus, thrombophlebitis, MRSA, mediastinitis, case report

## Abstract

**Introduction:**

Lemierre syndrome (LS) is a rare condition with a high mortality risk. It is well described in older children and young adults involving bacteremia, thrombophlebitis, and metastatic abscess commonly due to *Fusobacterium* infections. Young, pre-verbal children are also susceptible to LS; thus, careful attention must be given to their pattern of symptoms and history to identify this condition in the emergency department (ED).

**Case Report:**

A 12-month-old previously healthy boy with a recent diagnosis of acute otitis media and viral illness presented to the ED with a complaint of fever. Additional symptoms developed at the head and neck and were noted on subsequent ED visits. Advanced imaging revealed significant lymphadenopathy and deep space inflammation extending to the mediastinum. Subsequent imaging confirmed extensive sinus and deep vein thromboses, consistent with LS. Methicillin-resistant *Staphylococcus aureus* (MRSA) was the only organism identified. After surgical debridement, appropriate intravenous antibiotics, and heparin anticoagulation therapy, the patient experienced full recovery after prolonged hospitalization.

**Conclusion:**

A febrile infant with multiple acute care visits and development of lymphadenopathy, decreased oral intake, decreased cervical range of motion, and sepsis should raise suspicion for Lemierre syndrome. The medical evaluation of deep neck spaces and deep veins should be similar to that of older children and adults with LS, including advanced imaging of the head and neck. However, medical management should particularly target MRSA due to its emerging prevalence among infantile LS cases. Further research is necessary to determine the optimal management strategies of LS for this age group.

## INTRODUCTION

Lemierre syndrome (LS) is a well described condition characterized by the triad of internal jugular (IJ) thrombosis, pharyngitis, and metastatic abscess due to septic emboli. Patients classically present with fever and prolonged pharyngitis with a deteriorating clinical course. Historically, anaerobic pathogens, specifically *Fusobacterium necrophorum* or polymicrobial infections, cause LS.^1^ The median age of patients with LS is 22 years old.^2^ A minority of patients present within the first decade of life (8%).^2^

Although clinical diagnosis is typically made on signs and symptoms with computed tomography (CT) of the head and neck with intravenous (IV) contrast, it is critical to ultimately obtain cultures of the affected sites. Depending on the respective symptoms, imaging of other body areas should also be evaluated for metastatic abscesses. For example, there is documentation of septic emboli being found in the brain, lungs, and, as in this case, the mediastinum. Doppler ultrasound may enhance the assessment of thromboembolic disease.

Emergency management strategies emphasize cardiovascular resuscitation with IV fluids and broad-spectrum antibiotics, particularly when considering bacterial causes unique to LS. Early blood culture samples should be collected as they report positive in an estimated 86% of cases.^3^ The role of anticoagulation therapy for LS remains controversial.

## CASE REPORT

A 12-month-old boy presented to the emergency department (ED) with three days of fever. The mother was concerned for the development of right-sided facial swelling approximately three hours prior to presentation, increased irritability, and decreased oral intake. There were no reported gastrointestinal losses. The boy had an uncomplicated term birth and was vaccinated as appropriate for his age. Two weeks prior, he had completed a seven-day course of amoxicillin for acute otitis media (Figure). One day prior, the boy had visited a different ED with a complaint of “fever.” He had a urinalysis not suggestive of a urinary tract infection. After receiving antipyretics, he was discharged with supportive care.

**CPC-EM Capsule**

What do we already know about this clinical entity?

*Lemierre syndrome (LS) is a rare* Fusobacterium *bacteria oropharyngeal infection complicated by septic thrombophlebitis typically affecting adolescents and young adults.*

What makes this presentation of disease reportable?

*This case describes an infant with extensive LS with mediastinitis due to methicillin-resistant* Staphylococcus aureus *(MRSA).*

What is the major learning point?

*Young children are also at risk for LS, but the causative bacteria is more likely to be MRSA.*

How might this improve emergency medicine practice?

*Lemierre syndrome on the differential diagnosis for an infant with fever and lymphadenopathy will guide with antibiotic selection and understanding indication for neck computed tomography.*

On evaluation, the patient’s core temperature was 101.2° Fahrenheit (F), the peripheral pulse rate was 182 beats per minute, the respiratory rate was 36 breaths per minute, and pulse oximetry was 97%. He was a well-appearing and playful child in no acute distress and with slight right-sided facial swelling along the parotid region involving the mandible without fluctuance, crepitus, or cutaneous findings. His bilateral tympanic membranes were normal. His cardiorespiratory evaluation was grossly normal except for tachycardia, which was concordant with his fever. A soft-tissue head and neck ultrasound demonstrated bilateral submandibular lymph nodes, the largest measuring 2.7 × 1.5 × 1.5 centimeters (cm) on the right and 2.4 × 0.9 × 0.9 cm on the left without organized fluid collection, cavitation, or abscess. Such findings were presumed to be viral in etiology, and the patient was diagnosed with uncomplicated viral lymphadenitis and discharged with supportive management instructions.

The patient returned to the ED on his fourth day of illness with parental concern about worsening irritability, new avoidance of right-sided neck movement, and persistent fever. In addition, he had decreased oral intake without choking, vomiting, or respiratory distress, with resultant decreased urine output. His vital signs were as follows: temperature 102°F; blood pressure 118/75 millimeters of mercury, respirations between 28–35 breaths per minute, and pulse 140–156 beats per minute. His physical exam showed new and worsened right submandibular, submental, and cervical lymphadenopathy with decreased neck range of motion. However, he had a clear oropharyngeal exam and was protecting his airway. On otoscopy, he had a right-sided serous accumulation behind the tympanic membrane.

The basic metabolic panel was normal. His white blood cell count was 14.9 × 10^9^/microliters (μL) (reference range 5.5 × 10^9^/μL – 18 × 10^9^/μL), hemoglobin 10.2 grams per deciliter (g/dL) (10.5–13.5 g/dL), platelets 315 × 10^9^/μL (133 × 10^9^/μL – 450 × 10^9^/μL), with 72.2% neutrophils (15.0–40.0%). The whole blood lactic acid level was 1.5 millimoles per liter (mmol/L) (0.5–2.2 mmol/L), while procalcitonin was elevated at 0.78 nanograms ng/mL (normal high 0.10 ng/mL).

A chest radiograph was grossly unremarkable. Most notably, computed tomography (CT) of the neck and soft tissues with intravenous (IV) contrast revealed suppurative versus necrotic lymph nodes along the right upper neck, retropharyngeal phlegmonous/inflammatory changes, and fluid tracking inferiorly toward the mediastinum concerning for early mediastinitis (Image). Initial IV antibiotic management included vancomycin, cefepime, and metronidazole. The patient underwent a venous Doppler of the bilateral upper extremities in which the right IJ was not visualized. Complete echocardiography demonstrated a normal cardiac structure and no concerns for vegetations or pericardial effusion.

The patient was admitted to an inpatient step-down unit. Phlegmon and mediastinitis were managed operatively with incision and drainage by otorhinolaryngology (ENT). Wound cultures grew methicillin resistant *Staphylococcus aureus* (MRSA), and blood cultures demonstrated no growth.

Two days later, the patient acutely developed a worsening induration along the neck and a new fever, prompting another IV-contrast CT image of the neck and chest. Concerning findings included a right paratracheal abscess, supraclavicular and paratracheal lymphadenopathy, and an occlusive clot intracranially from the right sigmoid sinus that continued to the right IJ, right facial vein, and into the superior vena cava.

The patient went to the operating room emergently with ENT and cardiothoracic surgery for exploration. Trimethoprim/sulfamethoxazole was added to his care based on the recommendations of infectious diseases consultation. The team drained a purulent accumulation (5–7 mL) from the upper mediastinum and placed a transsternal, vacuum-assisted wound closure. Despite the extensive inflammation of surrounding structures, there was no cardiac or vascular involvement. The patient received multiple combinations of IV antibiotics during his hospitalization. Ultimately, the sensitivities of MRSA detection determined that vancomycin provided sufficient coverage.

A repeat venous Doppler ultrasound examination on the same day as his second surgery revealed a persistent lack of flow in the right IJ. The patient was started on heparin infusion and eventually transitioned to low-molecular-weight heparin with plans for a three-month anticoagulation therapy. Hypercoagulable workups did not identify other causes of thrombus.

Three months after discharge, a magnetic resonance venogram and Doppler ultrasound showed normal blood flow. Approximately eight months following his admission, he had a repeat CT chest with IV contrast and CT angiography of the head and neck that demonstrated full resolution of disease. Since then, the patient has not experienced any complications.

## DISCUSSION

Lemierre syndrome is a rare condition with high mortality. The condition is well described in older children and adults with classic presentation: sore throat or upper respiratory symptoms with prolonged fevers; lethargy; neck pain; lymphadenopathy; and sequelae of septic emboli. The presentation of this infant was consistent with LS: recent diagnosis of acute otitis media; lymphadenopathy; and persistently high fever. While pre-verbal children cannot describe pharyngeal pain, they commonly demonstrate it with decreased oral intake indicative of acute pharyngitis (e.g., herpangina or hand, foot, and mouth disease).^4^ Although lymphadenopathy of the head and neck in young children is common and often benign, this patient developed restricted neck mobility, which was concerning for occult deep space infection of the head and neck and prompted further workup. With appropriate imaging we identified diffuse inflammation and extensive thrombosis.

Few cases of LS have been reported in young children/infants. Like older patients, there are no known predisposing risk factors. Based on the findings of a previous systematic review, the top three sources of infection related to LS were the tonsils (37%), upper respiratory tract (30%), and chest/lower respiratory tract infections (25%).^1^ In this case, we believe that acute otitis media and an acute viral illness was the likely infectious source in accordance with what was described in previous medical evaluations. Head and neck infections, such as otitis media, are known to be associated with sigmoid sinus or other central venous thromboses, especially in preschool age children.^5^

*Fusobacterium necrophorum*, which is part of the normal anaerobic flora of the human oral cavity, is classically implicated as a cause of LS in older children and adults. However, a previous literature review reported that *Fusobacterium* infections in children <two years old are rare; only 12 cases in which *Fusobacterium* were the causative agents of mastoiditis have been documented during a 40-year period.^6^ Another retrospective review from 2017 documented six cases of LS secondary to MRSA in the pediatric population, a third of which were in infants <one year old.^7^ Hence, MRSA is an emerging source of LS, particularly in young children, and its preponderance coincides with the growing incidence of MRSA in the community since the early 2000s.^7^ In our patient, MRSA was the only pathogen isolated from cultures of suppurative mediastinal collections. Our findings have direct implications on the recommendations regarding initial antibiotic selection for LS in young children.

As in older subgroups, this child underwent a CT with contrast, which is the preferred diagnostic measure in LS to visualize the extent or complications of local inflammation and detect potential deep vein thromboses. He also received multiple Doppler ultrasounds to evaluate the extent of the IJ thrombus. Internal jugular and other deep vein thrombophlebitis is diagnostic for LS.^8^ However, the role of anticoagulation in preventing the extension of septic thrombi in LS remains controversial; some 64% of patient cases reported of LS received anticoagulation mostly as an adjunct therapy.^8^

The therapeutic emphasis for LS, however, is infection control over anticoagulation.^8^ To date, there are no guidelines for using anticoagulation in children with LS. Despite the initial surgical intervention targeting the source control and extensive clot burden, our patient’s clinical condition deteriorated. Therefore, a hematology consultant helped to guide the anticoagulation therapy recommendations for this patient, initially with heparin and then with low-molecular weight heparin. However, both short-term and long-term implications of treating young children with LS with anticoagulants are unknown.

Pediatric patients (66%) frequently require surgical interventions for the management of LS, which involve debridement, incision and drainage, and even exploratory laparotomy.^9^ This child was diagnosed with retropharyngeal phlegmon (14% of cases),^1^ abscess, and mediastinitis, and surgical interventions using ENT and cardiothoracic surgery were required after the clinical condition worsened.

## CONCLUSION

Diagnosis and management of Lemierre syndrome in young children necessitates a deep understanding of the various presentations and management strategies. One must pay astute attention to a constellation of subtle symptoms in presentation, including prolonged fever, lymphadenopathy, decreased oral intake, restricted range of neck motion, and multiple acute/subacute preceding healthcare visits. Rather than the traditional emphasis on anaerobic and polymicrobial antibiotic targets, the pathogen profile of young children with LS may require more emphasis on MRSA coverage.

Lemierre syndrome is a complex condition that is better understood and described in young adults and older children. Further investigation is needed to understand the predisposing factors for young children with LS and to employ optimal management strategies for the youngest age group affected by this condition.

## Figures and Tables

**Figure:**
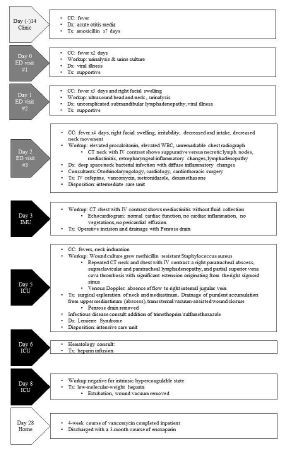
Timeline of events for an infant diagnosed with Lemierre syndrome. *ED*, emergency department; *IMU*, intermediate care unit; *ICU*, intensive care unit; *CC*, chief complaint; *Dx*, diagnosis; *Tx*, treatment; *WBC*, white blood cell; *CT*, computed tomography; *IV*, intravenous.

**Image:**
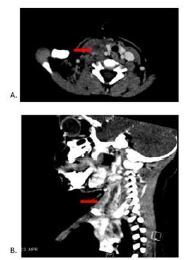
Computed tomography imaging of the neck with intravenous contrast shows multiple enlarged lymph nodes in the right upper neck with suppuration and/or necrosis; submandibular edema/phlegmonous change; mass effect of lymph nodes; fluid tracking inferiorly along the right neck extending to the superior mediastinum; and retropharyngeal phlegmonous/inflammatory changes (arrows). Right thrombophlebitis with occlusive clot beginning at the right sigmoid sinus to the superior vena cava. A) Axial view, B) sagittal view.

## References

[1] Karkos PD, Asrani S, Karkos CD (2009). Lemierre’s syndrome: a systematic review. Laryngoscope.

[2] Lee W-S, Jean S-S, Chen F-L (2020). Lemierre’s syndrome: a forgotten and re-emerging infection. J Microbiol Immunol Infect.

[3] Alves S, Stella L, Carvalho I (2019). Lemierre’s syndrome: a disguised threat. BMJ Case Rep.

[4] Amir J, Harel L, Smetana Z (1997). Treatment of herpes simplex gingivostomatitis with aciclovir in children: a randomised double blind placebo controlled study. BMJ.

[5] deVeber G, Booth F, Bjornson B (2001). Cerebral sinovenous thrombosis. New Engl J Med.

[6] Stergiopoulou T, Walsh TJ (2016). Fusobacterium necrophorum otitis and mastoiditis in infants and young toddlers. Eur J Clin Microbiol Infect Dis.

[7] Jariwala RH, Srialluri S, Huang MZ (2017). Methicillin-resistant *Staphylococcus aureus* as a cause of Lemierre’s syndrome. Pediatr Infect Dis J.

[8] Johannesen K, Bodtger U (2016). Lemierre’s syndrome: current perspectives on diagnosis and management. Infect Drug Resistance.

[9] Patel PN, Levi JR, Cohen MB (2020). Lemierre’s syndrome in the pediatric population: trends in disease presentation and management in literature. Int J Pediatr Otorhinolaryngol.

